# Editorial: Novel Techniques to Identify Immune Cell Population in Fish

**DOI:** 10.3389/fimmu.2022.893094

**Published:** 2022-04-12

**Authors:** Jia Cai, Jianmin Ye, Jorunn B. Jørgensen, Fumio Takizawa, Yasuhiro Shibasaki

**Affiliations:** ^1^ Fisheries College of Guangdong Ocean University, Southern Marine Science and Engineering Guangdong Laboratory (Zhanjiang), Guangdong Provincial Key Laboratory of Pathogenic Biology and Epidemiology for Aquatic Economic Animals & Key Laboratory of Control for Diseases of Aquatic Economic Animals of Guangdong Higher Education Institutes, Zhanjiang, China; ^2^ Guangdong Provincial Key Laboratory for Healthy and Safe Aquaculture, Guangdong Provincial Engineering Technology Research Center for Environmentally-Friendly Aquaculture, School of Life Sciences, South China Normal University, Guangzhou, China; ^3^ Universitetet i Tromsø (UiT) The Arctic University of Norway, Tromsø, Norway; ^4^ Faculty of Marine Science and Technology, Fukui Prefectural University, Obama, Japan; ^5^ College of Bioresource Sciences, Nihon University, Fujisawa, Japan

**Keywords:** fish, immune cell population, cell marker, activation and differentiation, immune system

As the first evolutionary group that comprises of innate immunity and adaptive immunity, fish is considered as a supreme model for clarifying the evolutionary and regulatory mechanisms of vertebrate immunity. However, the types and characteristics of fish immune cells are still not quite clear. In this Research Topic, eight articles including six original research articles and two review articles highlight the advance of novel techniques to identify immune cell population in fish.

The monoclonal antibody (mAb) specific for leukocyte surface markers is the classical approach to identify fish immune cells. Fei et al. reviewed the reagents (including mAbs of surface markers and immune cells) available for the research of fish immunity. The authors further discussed the potential applications of fluorescence-activated cell sorting and droplet-based microfluidics in screening and identifying antigen-specific B lymphocytes with a high-throughput manner and suggested to incorporate the alternative technologies to promote the production of specific antibodies in a high-throughput and cost-effective way. Similarly, the review article by Chan et al. summarized the protein marker and partial corresponding mAbs of teleost fish immune cells, and presented the interaction of fish T cells, B cells and dendritic cells *via* surface molecules for modulating adaptive immune response. More importantly, they further reviewed the advance for application of single-cell RNA sequencing (scRNA-seq) in teleost immunology and explored future directions of the methods developed for studying fish immunity at the cellular level. Martín et al. isolated two homologs of mammalian CD38 (CD38A and CD38B) from Rainbow trout (*Oncorhynchus mykiss*). By using the mAb against CD38A, CD38A^+^ populations among IgM^+^ B cells and IgM^-^ leukocytes of head kidney (HK) were screened *via* flow cytometry. The IgM^+^ CD38A^+^ B cells increased post-inactivated *Aeromonas salmonicida* stimulation *in vitro*, which produced higher levels of IgM and enhanced B cell differentiation gene transcription than the cells lacking CD38A.

The scRNA-seq is a newly developed technique that, also reviewed by Chan et al., already applied to identify previously unknown cell markers of teleost immune cell populations. In anterior kidney (AK) of Nile tilapia (*Oreochromis niloticus*), Wu et al. identified five distinct immune cell subsets including B cells, T cells, granulocytes, macrophages, and dendritic cells (DCs) and further uncovered different subsets of B-cell (pro/pre B cells, immature/mature B cells, activated B/plasmablasts, or plasma cells) and T cells (CD3^+^CD4^−^CD8^−^, CD3^+^CD4^+^CD8^+^, CD4^+^CD8^−^ and CD4^−^CD8^+^ T cells) based on distinct transcriptional level of the transcription factors (TFs) and cytokines. Additionally, Huang et al. analyzed scRNA-seq data of Orange-spotted grouper (*Epinephelus coioides*) with a full-length transcriptome as a reference, which was aimed to develop alternative approach for the fish samples without any published genome. In their study, four cell types including T cells, B cells, monocytes/macrophages (Mo/Mφ) and NCC (non-specific cytotoxic cells) were identified and two subsets of Mo/Mφ (M1 and M2 type), as well as four subsets in B cells (mature B cells, immature B cells, pre-B cells and early pre-B cells). Moreover, the finding of syngnathid fish, *Syngnathus typhle* (a fish species lacking the spleen and major histocompatibility class II (MHC II) pathway) by Parker et al. indicated that the loss of CD4^+^ T cells accompanied the loss of the MHC II pathwapresencehe present of regulatory T cells and cytotoxic T cells.

Although the scRNA-seq technique develops rapidly and contributes to the research field of fish immune cell, the analysis of transcriptome profile is still a reliable method to clarify the activity and development of specific immune cell type in fish. Smith et al. examined the mRNA transcriptome profiles at different stage of Atlantic salmon (*Salmo salar*) adherent head kidney leukocytes (HKLs) using microarray, and revealed that the adherent HKL cell population differentiates *in vitro* to become macrophage-like without exogenous stimulation, which might be regulated by miRNA and targeted differentially expressed genes (DEGs) associated with macrophage differentiation and function. Using transcriptome analysis, Park et al. revealed that the macrophage heterogeneity in adherent intestinal cells (AIC) and adherent head kidney cells (AKC), as well as the functional characteristics of mucosal and systemic macrophages in Atlantic salmon. Their data also suggested that interaction of miRNA and mRNAs related to macrophages and epithelial cells were involved in macrophage activation and differentiation.

In summary, all collected articles in this Research Topic exhibited recent novel works regarding fish immune cell identification and provided new strategy to better uncover the composition and function of teleost immune system, which can also help to expand our understanding for the evolution of the vertebrate immune system.

## Author Contributions

All authors contributed to this editorial insight and approved the submitted version.

## Funding

This work was supported by National Natural Science Foundation of China (U20A2065, 32073006, 31972818), Natural Science Foundation of Guangxi (2020GXNSFAA297243), Technology Planning Project of Guangdong Province of China (grant no. 2015A020209181), National Key R&D Program of China (2018YFD0900501), Independent Project of Southern Marine Science and Engineering Guangdong Laboratory (Zhanjiang) (No. ZJW-2019-06), Special Foundation for “Achieving the First Class” of Guangdong Province (231419013, 231419017), The Project of Guangxi Mangrove Coastal Wetland Ecological Protection and Sustainable Utilization Talent Highland (BGMRC202101), Guangdong South China Sea Key Laboratory of Aquaculture for Aquatic Economic Animals, Guangdong Ocean University (No. KFKT2019YB11), the Japan Society for the Promotion of Science KAKENHI Grants (JP20K06230, JP20K22594, JP20KK0144, JP21H02288).

## Conflict of Interest

The authors declare that the research was conducted in the absence of any commercial or financial relationships that could be construed as a potential conflict of interest.

## Publisher’s Note

All claims expressed in this article are solely those of the authors and do not necessarily represent those of their affiliated organizations, or those of the publisher, the editors and the reviewers. Any product that may be evaluated in this article, or claim that may be made by its manufacturer, is not guaranteed or endorsed by the publisher.

